# Ophthalmologic Implications of Connective Tissue Diseases: A Comprehensive Review of Current Knowledge and Innovations

**DOI:** 10.7759/cureus.67078

**Published:** 2024-08-17

**Authors:** Yuga B Pawar, Archana R Thool

**Affiliations:** 1 Department of Ophthalmology, Jawaharlal Nehru Medical College, Datta Meghe Institute of Higher Education and Research, Wardha, IND

**Keywords:** ophthalmologic innovations, sjögren's syndrome, systemic lupus erythematosus, rheumatoid arthritis, ocular manifestations, connective tissue diseases

## Abstract

Connective tissue diseases (CTDs), including rheumatoid arthritis (RA), systemic lupus erythematosus (SLE), Sjögren's syndrome, and systemic sclerosis (SSc), represent a diverse group of disorders characterized by abnormalities in the proteins that support tissues and organs. These diseases can affect multiple organ systems and are often associated with significant morbidity and mortality. The eyes are frequently affected among the various organ systems involved, with ocular manifestations ranging from benign conditions such as dry eye syndrome to severe, sight-threatening complications like scleritis, retinal vasculitis, and optic neuritis. Recognizing and managing these ophthalmologic implications is crucial for preventing severe complications, providing diagnostic clues, and improving patients' quality of life. This comprehensive review aims to elucidate the current knowledge and innovations related to the ophthalmologic implications of CTDs. It details the ocular manifestations associated with major CTDs, explores diagnostic approaches to identifying and differentiating these conditions, and discusses management strategies, including pharmacological and surgical interventions. Additionally, the review highlights recent advancements and emerging therapies in diagnosing and treating CTD-related ophthalmologic conditions. The review also addresses this field's challenges and future directions, emphasizing the importance of interdisciplinary collaboration and continuous research. By synthesizing the latest research and clinical insights, this review seeks to enhance the understanding of healthcare professionals regarding the interplay between CTDs and ocular health, ultimately contributing to improved patient care and outcomes.

## Introduction and background

Connective tissue diseases (CTDs) encompass a diverse group of disorders characterized by abnormalities in the proteins that support tissues and organs throughout the body [1]. These diseases include autoimmune conditions such as rheumatoid arthritis (RA), systemic lupus erythematosus (SLE), Sjögren's syndrome, and systemic sclerosis (SSc), among others [2]. CTDs can affect multiple organ systems, leading to a wide range of clinical manifestations and varying degrees of morbidity and mortality. The commonality among these diseases lies in their underlying pathophysiology, which often involves immune-mediated inflammation and tissue damage. For example, the immune system erroneously targets the synovial joints in RA, causing chronic inflammation and joint destruction. SLE is characterized by the production of autoantibodies that can affect virtually any organ system, resulting in a complex clinical presentation. Sjögren's syndrome primarily affects the exocrine glands, leading to dry eyes and mouth, while SSc is marked by excessive fibrosis and vascular abnormalities [3].

The eyes are frequently affected in CTDs, and ocular involvement can be among the earliest signs of these diseases. Recognizing and managing the ophthalmologic manifestations of CTDs is crucial for several reasons [4]. First, timely identification and treating ocular symptoms can prevent severe complications and preserve vision. Second, the presence of specific eye conditions may provide important diagnostic clues and help differentiate between different CTDs [5]. Third, ocular symptoms can significantly impact patients' quality of life, contributing to discomfort, visual impairment, and even blindness if left untreated. Ophthalmologic manifestations in CTDs can range from relatively benign conditions, such as dry eye syndrome, to more severe and potentially sight-threatening complications like scleritis, retinal vasculitis, and optic neuritis [6]. The complexity and variability of these manifestations necessitate a comprehensive understanding among healthcare providers to ensure optimal patient outcomes. Furthermore, advancements in diagnostic and therapeutic approaches offer new opportunities to improve the management of these conditions, making it imperative to stay updated with the latest developments in this field [6].

This review aims to provide a comprehensive overview of the current knowledge and innovations related to the ophthalmologic implications of CTDs. It will cover the following key areas: detailed descriptions of the ocular manifestations associated with major CTDs, including RA, SLE, Sjögren's syndrome, and SSc; diagnostic approaches to identifying and differentiating ophthalmologic conditions in patients with CTDs; management strategies, including pharmacological and surgical interventions, for treating ocular complications; recent advancements and emerging therapies in the diagnosis and treatment of CTD-related ophthalmologic conditions; and challenges and future directions in the field, with a focus on addressing treatment gaps and improving interdisciplinary collaboration. By synthesizing the latest research and clinical insights, this review aims to enhance the understanding of healthcare professionals regarding the interplay between CTDs and ocular health, ultimately contributing to better patient care and outcomes.

## Review

CTDs and their ophthalmologic manifestations

Rheumatoid Arthritis

RA is an autoimmune disease primarily affecting the joints, but it also has significant ocular implications that can severely impact patients' quality of life. One of RA's most common ocular manifestations is dry eye syndrome, known as keratoconjunctivitis sicca [7]. This condition results from inflammation and dysfunction of the lacrimal glands, leading to insufficient tear production. Symptoms of dry eye syndrome include a sensation of a foreign body, burning or itching, grittiness, light sensitivity, and blurred vision. Notably, a significant proportion of RA patients, estimated to be between 10% and 35%, may experience this condition. Additionally, up to 25% of RA patients may develop secondary Sjögren's syndrome, which can worsen dry eye symptoms and complicate treatment [8]. Another significant ocular complication linked to RA is scleritis and episcleritis. Scleritis, which affects the sclera, is more severe than episcleritis, which involves the episclera [9]. Scleritis occurs in approximately 4%-10% of RA patients and is characterized by symptoms such as redness, tenderness, light sensitivity, and intense pain. If left untreated, scleritis can lead to serious complications, including vision loss. Conversely, episcleritis is generally less severe, presenting with similar symptoms but typically resolving independently. Both conditions require careful monitoring and may necessitate treatment to manage inflammation and alleviate discomfort [10]. Corneal complications are also a concern for RA patients. These issues can arise from untreated dry eye syndrome, scleritis, or other inflammatory processes. Common corneal problems include superficial punctate keratitis, corneal ulceration, and corneal thinning [11]. Such complications can result in severe outcomes, including corneal melting and permanent vision loss, if not managed properly. Therefore, regular ophthalmologic evaluations are crucial for RA patients to monitor their eye health and address any emerging issues promptly [12].

Systemic Lupus Erythematosus

SLE is a complex autoimmune disease that can lead to various ocular manifestations, profoundly affecting patients' vision and quality of life. Key ocular complications associated with SLE include ocular surface disease, retinal vasculitis, and optic neuritis. Understanding these manifestations is crucial for timely diagnosis and effective management [13]. Ocular surface disease, particularly keratoconjunctivitis sicca, is the most common ocular manifestation in SLE, affecting approximately one-third of patients. This condition often results from secondary Sjögren's syndrome, characterized by reduced tear production, leading to significant discomfort and visual disturbances. Symptoms may include dryness, irritation, and a gritty eye sensation [14]. Histopathological findings in SLE patients with dry eyes may reveal loss of goblet cells and keratinization of the conjunctival epithelium, indicating chronic inflammation and damage to the ocular surface. Management strategies may involve using artificial tears, anti-inflammatory medications, and, in some cases, punctal plugs to improve tear retention [13]. Retinal vasculitis is a severe ocular complication of SLE and can result in significant vision loss. This condition is characterized by inflammation of the retinal blood vessels, which may present as cotton wool spots, retinal hemorrhages, and vascular sheathing. Lupus retinopathy affects approximately 3%-29% of SLE patients, particularly those with poorly controlled disease [15]. The pathophysiology involves immune complex deposition in the retinal vessels, leading to vasculitis and subsequent ischemia. Patients with antiphospholipid syndrome, which is common in SLE, are especially vulnerable to thrombotic events that can worsen retinal complications. Early detection and treatment with immunosuppressive therapy are critical to preventing permanent vision damage [16]. Optic neuritis, characterized by optic nerve inflammation, is another possible manifestation of SLE. It may present with symptoms such as vision loss, eye movement pain, and color perception changes. Although less common than other ocular manifestations, optic neuritis can significantly impact visual function and may require urgent intervention. Treatment typically involves high-dose corticosteroids to reduce inflammation and preserve vision. Prompt diagnosis and management are essential, as delayed treatment can lead to irreversible vision loss [17].

Sjögren's Syndrome

Sjögren's syndrome is a chronic autoimmune disorder primarily characterized by the dysfunction of exocrine glands, leading to significant dryness in the eyes and mouth. One of the hallmark ocular manifestations of this condition is keratoconjunctivitis sicca, commonly known as dry eye [18]. This results from lymphocytic infiltration of the lacrimal glands, causing decreased tear production. Patients typically experience symptoms such as burning, redness, light sensitivity, and visual disturbances. If left untreated, keratoconjunctivitis sicca can progress to more severe complications, including corneal ulceration, scarring, and even perforation, which can profoundly impact vision and quality of life [8]. Corneal involvement is another critical aspect of Sjögren's syndrome. Chronic dryness can lead to significant damage to the corneal epithelium, presenting as corneal abrasions and ulcers. These conditions increase the risk of infections and can further compromise vision. Effective management of corneal complications is essential, as they can result in long-term visual impairment. Patients with corneal involvement often require more intensive treatment and monitoring to prevent severe outcomes [19]. Additionally, individuals with Sjögren's syndrome are at an increased risk of developing lymphoma, particularly mucosa-associated lymphoid tissue lymphoma, in the conjunctiva [20]. This heightened risk is associated with chronic inflammation and lymphocytic infiltration, which are the characteristics of the syndrome. Regular monitoring for signs of conjunctival lymphoma is crucial, especially in patients with a long-standing history of Sjögren's syndrome or those exhibiting other risk factors. Early detection of malignancies can significantly improve outcomes and enable timely intervention [20].

Systemic Sclerosis

SSc, known as scleroderma, is a complex autoimmune disorder characterized by fibrosis, vasculopathy, and immune system dysregulation. This condition can lead to various ocular manifestations, which clinicians must recognize for early diagnosis and effective management. The ophthalmic implications of SSc include keratoconjunctivitis sicca, eyelid skin changes, retinal vasculopathy, cataracts, and glaucoma [21]. One of the most common ocular manifestations in patients with SSc is keratoconjunctivitis sicca, or dry eyes, which affects nearly 49% of individuals with the disease. This condition is often linked to the underlying autoimmune process and can significantly impact patients' quality of life. Additionally, fibrosis of the eyelid skin is prevalent, with over 50% of SSc patients exhibiting changes that can lead to cosmetic concerns and functional issues, such as difficulty in eyelid closure. These alterations can exacerbate discomfort and dryness, worsening the symptoms of keratoconjunctivitis sicca [22]. Retinal vasculopathy is another significant ocular complication associated with SSc. This condition is characterized by alterations in the retinal microcirculation, which may resemble findings seen in systemic hypertension [23]. Approximately 28.9% of SSc patients show retinal microvascular abnormalities, indicating potential vascular involvement. These changes can lead to complications such as retinal ischemia, and their presence may correlate with disease severity, serving as a marker for systemic involvement [24]. Cataracts and glaucoma are also significant concerns for patients with SSc. The prevalence of cataracts in SSc patients is notable, with rates reported around 42.2% [25]. While some cataracts may be age-related, others could be secondary to corticosteroid treatments commonly used in managing SSc. The exact mechanisms linking SSc to cataract formation remain unclear, but chronic inflammation and oxidative stress are likely contributing factors [25]. Similarly, glaucoma has been reported in approximately 13.3% of SSc patients. The pathophysiology may relate to changes in intraocular pressure and vascular supply to the optic nerve, although further research is needed to establish a clear connection [26].

Diagnostic approaches

**Figure 1:**
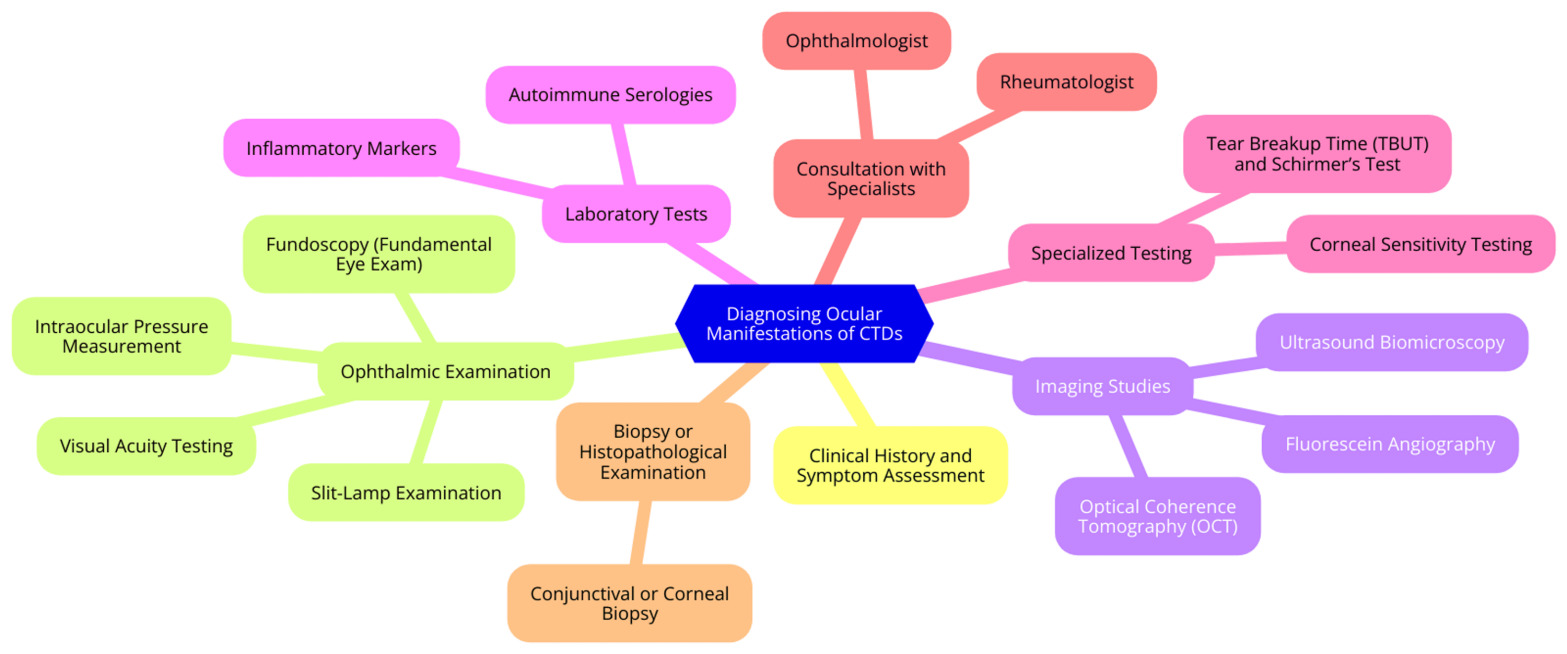
Diagnosing ocular manifestations of CTDs Image credit: Yuga Pawar CTDs: connective tissue diseases

Diagnosing ocular manifestations of CTDs requires a comprehensive approach integrating clinical evaluation techniques, advanced imaging modalities, and laboratory tests. This multifaceted strategy is essential for ensuring accurate diagnosis and effective management of ocular complications associated with these complex disorders [5]. Clinical evaluation begins with a detailed patient history, where healthcare providers inquire about symptoms such as dryness, redness, pain, and visual disturbances. It is also essential to assess systemic symptoms related to CTDs, such as joint pain or skin rashes [27]. A thorough ocular examination follows, including visual acuity testing to establish a baseline and a slit-lamp examination to evaluate the anterior segment of the eye. This examination is crucial for identifying conditions like scleritis, keratitis, and dry eye syndrome. Additionally, fundoscopy allows for the evaluation of the retina and optic nerve head, helping to detect signs of uveitis or other retinal complications [27]. Advanced imaging modalities play a significant role in the diagnostic process. Optical coherence tomography is a noninvasive technique that provides high-resolution cross-sectional retina and optic nerve images. It is invaluable for detecting retinal changes such as edema and atrophy associated with uveitis and other inflammatory conditions [28]. Fluorescein angiography, which involves injecting fluorescein dye to visualize blood flow in the retina, is particularly useful for identifying retinal vascular changes, such as leakage or occlusion, which can occur in conditions like lupus or RA. Additionally, B-scan ultrasonography can assess posterior segment issues, including retinal detachment or vitreous hemorrhage, especially in severe scleritis or uveitis [29]. Laboratory tests and biomarkers further enhance the diagnostic process. An autoimmune panel that tests for specific autoantibodies, such as antinuclear antibodies, rheumatoid factor, and anti-double-stranded DNA antibodies, can help confirm the diagnosis of underlying CTDs [30]. Inflammatory markers like C-reactive protein and erythrocyte sedimentation rate can indicate systemic inflammation, which may correlate with ocular inflammation. Additionally, tear film analysis, including the Schirmer test and tear film osmolarity testing, can provide insights into the severity of dry eye disease, a common complication in CTDs. In some cases, genetic testing may be considered, mainly if there is a family history or atypical presentation [31]. Diagnosing ocular manifestations of CTDs are shown in Figure 1.

Management strategies

Managing the ocular manifestations of CTDs requires a comprehensive approach that integrates pharmacological and surgical strategies. These strategies are crucial for addressing these conditions' diverse and often complex symptoms [5]. Pharmacological interventions are fundamental in managing ocular symptoms related to CTDs. Immunomodulators, such as methotrexate and azathioprine, are commonly used to reduce inflammation and control autoimmune responses. Methotrexate, for example, is particularly effective in conditions like RA, where it can help prevent ocular complications [32]. The advent of biologics has marked a significant advancement in treatment. Tumor necrosis factor-alpha inhibitors, such as infliximab and adalimumab, effectively treat scleritis and other inflammatory eye conditions. Interleukin-6 inhibitors, such as tocilizumab, can also benefit patients with severe inflammatory eye disease, especially RA. B-cell depletion therapies, like rituximab, may be considered for refractory cases, particularly in patients with SLE [33]. Topical treatments are essential for managing ocular surface symptoms, such as dry eye, which is common in CTD patients. Artificial tears are the primary treatment for providing lubrication and relief. Anti-inflammatory drops, including corticosteroids, may be prescribed for short-term inflammation management related to conditions such as scleritis or severe dry eye [34]. Cyclosporine A (Restasis) is another effective option for enhancing tear production in patients with chronic dry eye. In more severe cases, punctal plugs can occlude the tear ducts, helping retain tears on the ocular surface and alleviate symptoms [34]. Surgical interventions may be necessary for managing severe ocular complications arising from CTDs. Cataract surgery, for instance, requires careful timing and control of the underlying disease to minimize complications. Surgeons must also consider potential issues related to corneal thinning or scleral fragility in these patients, necessitating a thorough preoperative assessment [35]. Scleral grafting may be indicated in cases of severe scleritis or corneal perforation. This procedure involves placing a donor scleral patch to reinforce weakened areas, restoring ocular integrity and preventing vision loss. Corneal transplantation, or penetrating keratoplasty, may be required for patients with significant corneal scarring or thinning due to CTDs. Postoperative monitoring is essential to manage potential graft rejection. Additionally, surgical interventions such as vitrectomy may be necessary for patients with retinal detachment or severe retinal vasculitis, requiring a multidisciplinary approach to achieve optimal outcomes [36].

Innovations and emerging therapies

Recent advancements in diagnostic technology and emerging therapies profoundly transform healthcare, particularly in managing various diseases. Innovations in diagnostic methods, novel treatment approaches, gene therapy, and regenerative medicine applications are reshaping our understanding and treatment of medical conditions [37]. One of the most notable advancements in diagnostics is the rise of molecular diagnostics. Techniques such as polymerase chain reaction and next-generation sequencing have significantly improved the sensitivity and specificity of disease detection, enabling rapid identification of pathogens and genetic mutations. This precision allows for treatments tailored to individual patients [38]. Moreover, artificial intelligence and machine learning are increasingly integrated into diagnostic processes, enhancing clinical decision support systems. These technologies analyze complex patient data, identify patterns in large datasets, and lead to more accurate diagnoses and better patient outcomes [38]. Point-of-care testing devices are revolutionizing diagnostics by providing immediate results at the patient's location. This capability facilitates timely treatment decisions, particularly in acute care settings. Telemedicine and wearable technology are also advancing continuous health monitoring and remote consultations. This patient-centered approach enhances accessibility and convenience, providing more timely care [39]. In treatment, biological agents are becoming more prevalent in managing autoimmune diseases and cancers. These agents, including monoclonal antibodies and recombinant proteins, target specific disease pathways, offering personalized treatment options. The application of mRNA technology, initially popularized by COVID-19 vaccines, is expanding into diagnostics and therapeutics. This innovative technology enables the rapid development of targeted treatments and diagnostics by instructing cells to produce specific proteins, thus enhancing the body’s immune response [40]. Nanomedicine represents another exciting development, exploring the potential of nanotechnology in drug delivery systems and imaging techniques. For example, gold nanoparticles can enhance cancer imaging, improve tumor detection accuracy, and potentially lead to better treatment outcomes. These novel approaches signify a shift toward more effective, personalized healthcare solutions [41]. Gene therapy is a groundbreaking advancement in treating genetic disorders. Technologies like clustered regularly interspaced short palindromic repeats pave the way for innovative therapies to correct defective genes responsible for disease development, offering potential cures for previously untreatable conditions. This approach holds promise for genetic diseases and conditions like cancer, where targeted gene editing can disrupt malignant cell growth [42]. Regenerative medicine is making significant strides in repairing or replacing damaged tissues and organs. Techniques such as 3D bioprinting are being explored to create functional tissues, which could revolutionize transplantation and personalized medicine. Stem cell therapies are also being developed to treat various conditions, including degenerative diseases and injuries. By harnessing the body’s ability to regenerate damaged tissues, these therapies offer new avenues for treatment and recovery [43].

Challenges and future directions

Addressing the challenges and future directions in managing the ophthalmic implications of CTDs requires a comprehensive strategy that includes identifying treatment gaps, enhancing interdisciplinary collaboration, and outlining future research priorities [44]. One of the primary challenges is the presence of significant treatment gaps. Despite advances in understanding CTDs and their ocular manifestations, many patients face delays in diagnosis and management due to the variability in symptoms and the complexity of these disorders. Improved screening protocols are essential for the early identification of ocular manifestations, which can often precede systemic symptoms [45]. Regular eye examinations should be incorporated into the management plans for patients with known CTDs to facilitate early intervention. Additionally, disparities in access to specialized care can hinder effective management. Raising awareness among healthcare providers about the ocular implications of CTDs can improve referrals and treatment strategies, ultimately enhancing patient outcomes [45]. Another critical aspect is the need for better interdisciplinary collaboration. Establishing multidisciplinary teams that include rheumatologists, ophthalmologists, and other specialists is crucial for optimal management of CTDs and their ocular implications. Such teams can develop comprehensive management plans addressing systemic and ocular symptoms, ensuring a holistic approach to patient care. Enhancing communication between different specialties and educating healthcare providers about the ocular manifestations of CTDs can lead to more timely referrals and interventions. Regular case discussions and shared clinical pathways can foster a collaborative environment, benefiting patient care [46]. Future research in CTD-related ophthalmology should focus on several key areas to enhance understanding and management of these conditions. Longitudinal studies are needed to elucidate the progression of ocular manifestations in CTD patients, identify risk factors, and improve management strategies [5]. Additionally, research into innovative treatment modalities, including biologics and targeted therapies, holds promise for more effective management of ocular symptoms. Investigating the efficacy of these treatments in the context of CTDs could lead to significant advancements in care. Future studies should also emphasize patient-centered approaches, focusing on patient-reported outcomes and quality-of-life assessments. Understanding the impact of ocular symptoms on daily living and overall health in CTD patients is essential for developing effective management strategies [5].

## Conclusions

Understanding the ophthalmologic implications of CTDs is crucial for effectively managing and treating affected patients. CTDs such as RA, SLE, Sjögren's syndrome, and SSc can have significant ocular manifestations that, if not promptly identified and managed, can lead to severe complications and diminished quality of life. This comprehensive review has highlighted the diverse range of eye conditions associated with these diseases, the importance of early and accurate diagnosis, and the various management strategies available, including pharmacological and surgical interventions. Advances in diagnostic technology and emerging therapies offer hope for improved patient outcomes. However, challenges remain, particularly in addressing treatment gaps and fostering interdisciplinary collaboration. Future research should focus on these areas to enhance our understanding and treatment of CTD-related ophthalmologic conditions. By staying informed about the latest developments and innovations, healthcare providers can ensure better care for patients with these complex diseases, ultimately improving their ocular health and overall well-being.
